# Development and validation of an interpretable radiomic signature for preoperative estimation of tumor mutational burden in lung adenocarcinoma

**DOI:** 10.3389/fgene.2024.1367434

**Published:** 2024-04-10

**Authors:** Yuwei Zhang, Yichen Yang, Yue Ma, Ying Liu, Zhaoxiang Ye

**Affiliations:** ^1^ Department of Radiology, Tianjin Medical University Cancer Institute and Hospital, National Clinical Research Center for Cancer, Tianjin’s Clinical Research Center for Cancer, Key Laboratory of Cancer Prevention and Therapy of Tianjin, Key Laboratory of Cancer Immunology and Biotherapy of Tianjin, Tianjin, China; ^2^ Department of Epidemiology and Biostatistics, Tianjin Medical University Cancer Institute and Hospital, National Clinical Research Center for Cancer, Tianjin’s Clinical Research Center of Cancer, Key Laboratory of Cancer Prevention and Therapy of Tianjin, Tianjin, China

**Keywords:** radiomics, tumor mutational burden, machine learning, lung adenocarcinoma, immunotherapy biomarker

## Abstract

**Background:**

Tumor mutational burden (TMB) is a promising biomarker for immunotherapy. The challenge of spatial and temporal heterogeneity and high costs weaken its power in clinical routine. The aim of this study is to estimate TMB preoperatively using a volumetric CT–based radiomic signature (rMB).

**Methods:**

Seventy-one patients with resectable lung adenocarcinoma (LUAD) who underwent whole-exome sequencing (WXS) from 2011 to 2014 were enrolled from the institutional biobank of Tianjin Medical University Cancer Institute and Hospital (TMUCIH). Forty-nine LUAD patients with WXS from the Cancer Genome Atlas Program (TCGA) served as the external validation cohort. Computed tomography (CT) volumes were resampled to 1-mm isotropic, semi-automatically segmented, and manually adjusted by two radiologists. A total of 3,108 radiomic features were extracted via PyRadiomics and then harmonized across cohorts by ComBat. Features with inter-segmentation intra-class correlation coefficient (ICC) > 0.8, low collinearity, and significant univariate power were passed to the least absolute shrinkage and selection operator (LASSO)–logistic classifier to discriminate TMB-high/TMB-low at a threshold of 10 mut/Mb. The receiver operating characteristic (ROC) curve analysis and calibration curve were used to determine its efficiency. Shapley values (SHAP) attributed individual predictions to feature contributions. Clinical variables and circulating biomarkers were collected to find potential associations with TMB and rMB.

**Results:**

The top frequently mutated genes significantly differed between the Chinese and TCGA cohorts, with a median TMB of 2.20 and 3.46 mut/Mb and 15 (21.12%) and 9 (18.37%) cases of TMB-high, respectively. After dimensionality reduction, rMB comprised 21 features, which reached an AUC of 0.895 (sensitivity = 0.867, specificity = 0.875, and accuracy = 0.873) in the discovery cohort and 0.878 (sensitivity = 1.0, specificity = 0.825, and accuracy = 0.857 in a consist cutoff) in the validation cohort. rMB of TMB-high patients was significantly higher than rMB of TMB-low patients in both cohorts (*p* < 0.01). rMB was well-calibrated in the discovery cohort and validation cohort (*p* = 0.27 and 0.74, respectively). The square-filtered gray-level concurrence matrix (GLCM) correlation was of significant importance in prediction. The proportion of circulating monocytes and the monocyte-to-lymphocyte ratio were associated with TMB, whereas the circulating neutrophils and lymphocyte percentage, original and derived neutrophil-to-lymphocyte ratio, and platelet-to-lymphocyte ratio were associated with rMB.

**Conclusion:**

rMB, an intra-tumor radiomic signature, could predict lung adenocarcinoma patients with higher TMB. Insights from the Shapley values may enhance persuasiveness of the purposed signature for further clinical application. rMB could become a promising tool to triage patients who might benefit from a next-generation sequencing test.

## 1 Introduction

Immune checkpoint inhibitors targeting programmed death-1 (PD-1) or its ligand (PD-L1) have come up on the stage of first-line treatment in non–small-cell lung cancer (NSCLC). Favorable improvement on survival outcomes has been observed in both metastatic and resectable populations and enhanced in non-squamous NSCLC. Nevertheless, an estimated objective response rate of 26.91% in a pooled meta-analysis has spoken yet again of the necessity for precise beneficiary selection (Chen et al., 2021). To this end, exploration in predictive biomarkers for immune checkpoint inhibitors has never stopped. The first United States Food and Drug Administration (FDA)-approved biomarker for checkpoint inhibitors is the expression level of PD-L1, defined by positive staining of tumor cytomembrane on immunohistochemistry (IHC) slides, which directly regulates the adaptive anti-tumor immune response (Doroshow et al., 2021). It has been confirmed effective but imperfect for the decision of offering immunotherapy because it is insufficient to explain the benefits of patients with a PD-L1 tumor proportion score (TPS) <50%, which might be owing to the heterogeneity of tumor microenvironments and other technical factors (Shen and Zhao, 2018). In addition, predictive efficiency of PD-L1 expression varies across histopathological subtypes of NSCLC. A retrospective study revealed that patients with non-squamous NSCLC and higher PD-L1 expression were more likely to benefit from mono- or dual-immune checkpoint inhibitors (Meshulami et al., 2023).

Subsequently, the FDA has approved tumor mutational burden (TMB), which measures the number of somatic mutations per megabase of specific cancer genomic sequences (Sha et al., 2020) as the second pan-cancer companion diagnostics at a threshold of 10 mut/Mb for PD-1 inhibitors after microsatellite instability or deficient mismatch repair. TMB is convinced to be a snapshot of the evolutionary complexity in cancer genome and the pivotal source of neoantigens that contribute to tumor-specific T-cell response in tumor microenvironments (Jia et al., 2018), and then eventually shapes the individual response to immune checkpoint inhibitors (Rizvi et al., 2015). Evidence from Checkmate-026 trail has suggested that TMB can identify a subgroup that may benefit from PD-1 inhibitors among NSCLC patients with PD-L1 expression levels ≥5% (Carbone et al., 2017). A multi-center cohort study has revealed that TMB-high outperformed PD-L1 in predicting the response and survival outcomes of NSCLC patients who received PD-L1 inhibitors that were associated with higher infiltrating CD8^+^ T cells and upregulations of several immune-related signaling pathways (Ricciuti et al., 2022). In a recent real-world study, elevated TMB (≥10 mut/Mb) was confirmed to be associated with durable benefit on checkpoint inhibitors across various cancer types (Gandara et al., 2023). Nonetheless, there still remains challenges in the application of TMB. First of all, TMB in lung adenocarcinoma is significantly lower than that in squamous cell carcinoma, which may require a larger panel, coverage, and depth to capture enough signals of nucleotide variations. Second, it could be affected by temporal and spatial heterogeneity of tumor as well; hence, single sample–based TMB estimation is not recommended (Kazdal et al., 2019; Stein et al., 2019). In clinical practice, the use of biopsy samples may magnify such an effect that results in over- or underestimation of TMB. Furthermore, despite next-generation sequencing (NGS) and panel-based targeting sequencing substantially reducing the cost of genomic assessment, testing TMB is still more expensive than that of immunohistochemistry-based biomarkers. As a consequence, there is still a need for developing non-invasive, comprehensive, and accurate diagnostic frameworks to expand the application and value of TMB.

Radiomics, a machine learning-enabled high-throughput characterization of images, has established robust and convincing relations among imaging phenotypes, clusters of molecular phenotypes, and genotypes in NSCLC (Wu et al., 2022). It takes the advantages of imaging scans that globally, dynamically present the landscape of *in vivo* heterogeneity as a part of the standard-of-care procedures in cancer diagnosis, staging, and monitoring of therapeutic effects (Bi et al., 2019). Heretofore, there exists sufficient evidence that confirms imaging phenotypes, from radiologic semantics to deep learning-encoded radiomic signatures, which are capable of predicting specific driver mutations in NSCLC. Liu et al. have reported the association between CT semantic features and the epidermal growth factor receptor (EGFR) genotype (Liu et al., 2016). A bulk of radiomic signatures that have integrated both intra-tumor and peritumor information were successfully constructed to predict the mutational status of the EGFR (Rios Velazquez et al., 2017; Shang et al., 2023). The latest international large-scale multi-cohort study enrolled 18,232 patients to further validate the efficiency of CT-based whole-lung biomarkers to recognize the EGFR genotype and risk of resistance to tyrosine kinase inhibitors (Wang et al., 2022). However, insights that expand the cross-scale relevance to mutational loads of the whole genome are still limited. A plausible association has been reported between CT semantics (Zhang et al., 2020) and radiomic signatures (Yang et al., 2023) without the constant threshold of TMB and interracial validation.

To this end, the current study purposes to develop and validate an interpretable CT-based radiomic signature, radiological mutational burden (rMB), which is capable of discriminating lung adenocarcinoma between dichotomous TMB levels to triage patients who are most likely to benefit from sequencing and immune checkpoint inhibitors.

## 2 Materials and methods

This retrospective study was conducted in accordance with the Declaration of Helsinki and approved by the institutional ethics committee (Approval ID. Ek2021067). Informed consent was signed to authorize the storage and further investigation of tissue samples from each participant.

### 2.1 Patients

The TMUCIH-LUAD cohort, as the discovery cohort, comprised patients who received surgical resection of primary lung adenocarcinoma and authorized the storage of their samples in the institutional biobank from 1 January 2011 to 1 January 2014. The primary eligibility criteria included patients who had a) received at least a wedge resection with systematic lymph nodes dissection; b) received pathological confirmation of lung adenocarcinoma; c) deposited paired tumor and control sample in the institutional biobank; and d) completed preoperative CT scan 30 days before surgery. The exclusion criteria included a) significant DNA degradation or pollution of sample caused by proteins or RNA, which may cause failure in library preparation; b) unavailable or expired preoperative radiological studies in the picture archiving and communication system; c) untraceable data from electronic medical record or any disagreement in answering queries when collecting clinical and pathological data.

A subset of the TCGA-LUAD cohort was included in this study for externally validating the proposed rMB (www.cancerimagingarchive.net/collection/tcga-luad) from the cancer imaging archive (TCIA). After matching the radiological studies from the TCIA with the available genomic profiles from the Genomic Data Commons (GDC, portal.gdc.cancer.gov), a further exclusion of data was performed according to the following criteria: studies without a CT modality (Chen et al., 2021); the lack of preoperative scan (Doroshow et al., 2021); and poor image quality induced by mental implants or motion (Shen and Zhao, 2018).

### 2.2 Clinical data

Owing to the limited demographic and clinical information in the TCGA-LUAD, eight baseline variables were collected and aligned: age, sex, side and lobe of primary tumor, attenuation, and the TNM stages according to the eighth edition of the American Joint Committee on Cancer TNM staging system. In the TMUCIH-LUAD cohort, the TNM staging was retrospectively collected from pathological reports, whereas it was either edited from existing staging variables or manually evaluated according to the radiological profiles in the TCIA if absent in the original TCGA-LUAD database. For cases with multiple lesions, the T-stage was determined by the tumor resected for WXS sequencing.

In the TMUCIH-LUAD cohort, smoking history, pack-year smoked grading, alcohol exposure, family history of malignancy, and history of prior or synchronous malignancy were collected as supplement to further discover the latent association between rMB and TMB-related clinical variables. In addition to the three serum tumor markers: carcinoembryonic antigen (CEA), neuron-specific enolase (NSE), and tissue polypeptide–specific antigen (TPSA), the percentage of circulating neutrophils, lymphocytes, monocytes, and six derived inflammatory biomarkers that included the neutrophil to lymphocyte ratio (NLR, absolute neutrophil count/absolute lymphocyte count), derived NLR (dNLR, absolute neutrophil count/the difference of absolute white cell count and neutrophil count), platelet-to-lymphocyte ratio (PLR, absolute platelet count/absolute lymphocyte count), monocyte-to-lymphocyte ratio (MLR, absolute monocyte count/absolute lymphocyte count), systemic immune-inflammation index (SII, absolute platelet count × NLR), and serum lactate dehydrogenase (LDH) were also recorded from the laboratory information system to probe the immune relevant of rMB.

### 2.3 Genomic profiling and TMB calculation

For the TMUCIH-LUAD cohort, a commercial whole-exome target enrichment system (SureSelect^XT^ V6, Agilent Technologies) was utilized to perform the NGS test (Illumina HiSeq 2500 platform) with purified DNA samples that were isolated from formalin-fixed paraffin-embedded tumor slices. Normal lung tissue from the same surgical specimen or 2–5 mL of blood sample stored in liquid nitrogen was paired as the control sample. Somatic mutations were called by the Mutect2 algorithm using reference genome GRCh37 and then filtered. For the TCGA-LUAD cohort, an ensemble of aliquot-level mutational landscape of each sample was downloaded from the GDC. TMB was defined as the sum of somatic mutations divided by the capture size of the coding base, which was set to 35.8 Mb in this study. A cut-off value of 10 mut/Mb, as approved by the FDA, dichotomized TMB into two levels: TMB-low and TMB-high.

### 2.4 CT image acquisition and segmentation

For the TMUCIH-LUAD cohort, CT data were obtained from four scanners (Discovery ST, Discovery 750HD, Lightspeed 16 from General Electric Healthcare, Boston, Massachusetts, USA; SOMATOM Definition AS+ from Siemens, Erlangen, Germany) with a tube voltage of 120–140 kVp, automatic tube current, and a field of view of 40 cm. The images were reconstructed in a matrix of 512 × 512 pixels, with slice thicknesses of 1.25 mm and 1.5 mm for scanners from two vendors, respectively, without any overlapping between the slices. For the TCGA-LUAD cohort, the scanning and reconstruction parameters varied across patients, with a tube voltage of 120–140 kVp, automatic tube current, and a unified matrix of 512 × 512 pixels.

The original CT slices were resampled to 1 mm isotropic volumes via B-spine interpolation, then segmented by a radiologist with 5 years' experience in thoracic imaging. The contour of the gross tumor volume was initialized by the active contour mode in ITK-SNAP (version 4.0.2, www.itksnap.org). First, a bounding box that completely covered the lesion within a proper interval of CT-value was manually initiated to avoid the spatial or gray-level overflow of the contour; next, active bubbles were randomly placed in the lesion, which then automatically grew together with proper force of smoothing and region competition; finally, segmentation was adjusted along the edges of the lesion, slice-by-slice to ensure accuracy. An additional test–retest subset, which comprised 30 volumes that were randomly sampled from the TMUCIH-LUAD cohort, was re-segmented in the same fashion by another radiologist, for evaluating the reproducibility of radiomic features. The DICE coefficient was calculated to measure the similarity between the gross tumor volumes from the two radiologists.

### 2.5 Development and validation of rMB

A total of 3,108 radiomic features were extracted on the PyRadiomics platform (version 3.0.1). Initially, features with near-zero variance were removed prior to further processing. Then, the ICC was calculated to measure the consistency of feature values against the variations of contour using the test–retest subset, where features with ICC < 0.8 were removed. Next, ComBat harmonization was applied to compensate cross-vendor and cross-protocol variations on the feature scale, where the batch effect was encoded into seven unique identifiers according to the combination of the original slice thickness, types of convolution kernels, and application of the contrast agent. A spreadsheet for detailed scanning parameters and their ComBat unique identifiers were presented in the Supplementary Material 1.

Feature selection was divided into three steps and was all applied in the training set: first, the Spearman correlation coefficients were calculated to filter the features that were irrelevant to TMB at the threshold of 0.2. Then, collinearity between the features was diagnosed iteratively by using the matrix of Pearson correlation in which features with r ≥ 0.9 were regarded collinear, and then, the one with the smaller mean absolute correlation was to be kept. Eventually, univariate negative binomial regression and the Mann–Whitney *U* test were used together to identify the final set of features associated with continuous TMB and to categorize the TMB levels.

To develop rMB associated with the TMB levels, a logistic classifier with LASSO-selected features was established after optimizing the hyper-parameter λ by minimizing the area-under-the-curve (AUC) error through 10-fold cross-validation, which gradually increased L1-norm penalties to coefficients and thereby resulted in sparsity of feature weights. The ROC curves were illustrated to diagnose the performance of rMB in the development and validation cohorts. A comparison of rMB between the TMB levels was made to diagnose discrimination, and calibration curves with the Hosmer–Lemeshow test were utilized to evaluate calibration subsequently. Shapley values attributed individual predictions to feature contributions for *post hoc* interpretation of LASSO–logistic classifier.

### 2.6 Statistical analysis

All machine learning pipelines and statistical analyses were conducted in R version 4.3.2 (https://cran.r-project.org/src/base/R-4/). Any two-tailed *p*-value < 0.05 was regarded as statistically significant. Comparisons of categorical variables and frequencies of mutated genes between groups and cohorts were made via the chi-squared test or Fisher’s exact test. The Shapiro–Wilk test was used to examine whether the continuous variables followed a normal distribution at each level. The Student’s or Welch *t*-test and Mann–Whitney *U* test were used for continuous variables according to the normality and variances of two samples. A comparison between the AUCs was examined by using the DeLong’s test. Associations between rMB, TMB levels, and clinical laboratory variables were assessed by using the univariate linear and logistic regression. The source code for each figure was provided in the Supplementary Material 2.

## 3 Results

### 3.1 Patients and mutational landscapes

**FIGURE 1:**
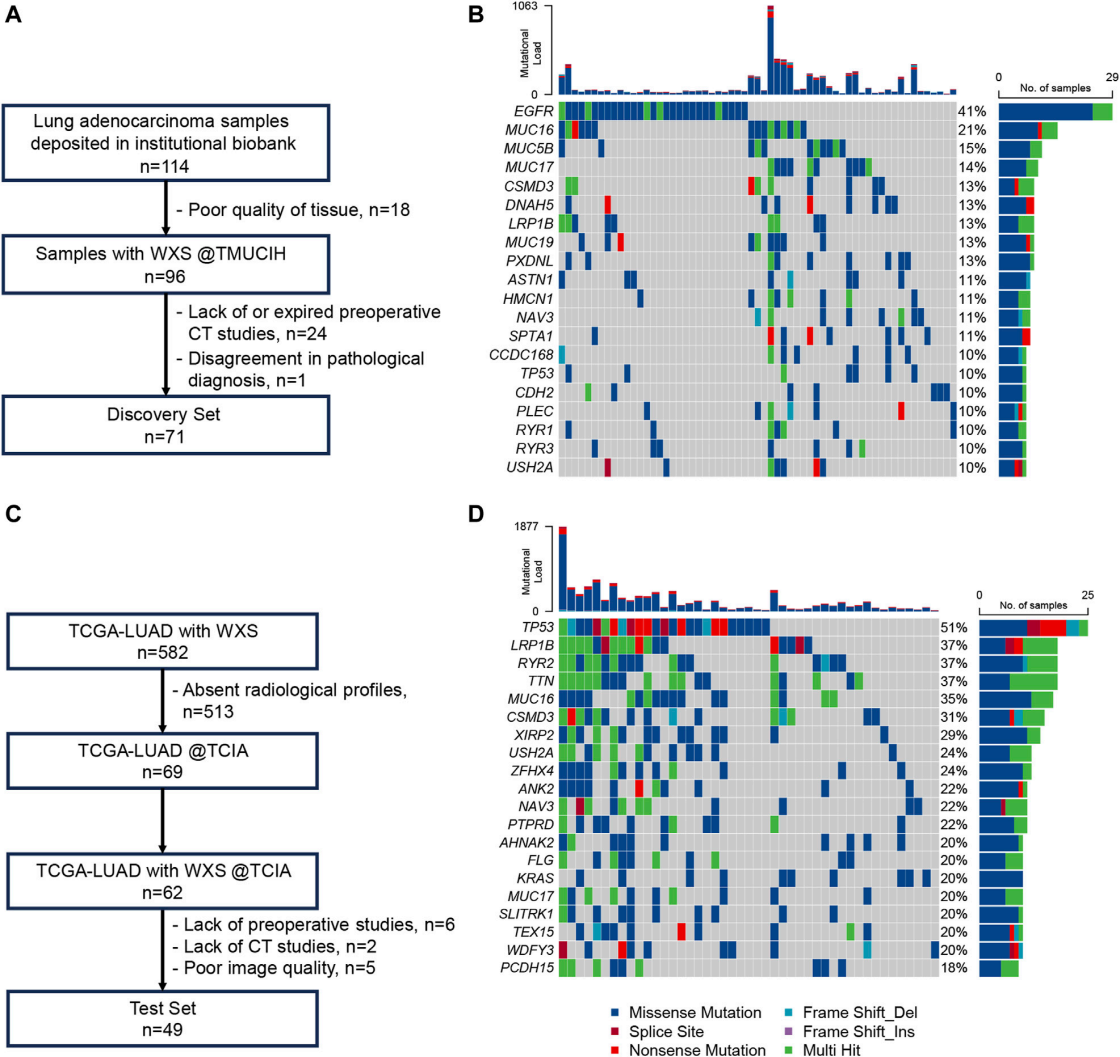
Patients and genomic landscapes. **(A)** Diagram of patient inclusion and exclusion in TMUCIH-LUAD; **(B)** genomic landscape of the top 20 frequently mutated genes in TMUCIH-LUAD; **(C)** diagram of patient inclusion and exclusion in TCGA-LUAD; **(D)** genomic landscape of the top 20 frequently mutated genes in TCGA-LUAD.

The TMUCIH-LUAD and TCGA-LUAD cohorts comprised 71 and 49 LUAD patients with a median TMB of 2.2 mut/Mb and 3.5 mut/Mb, respectively. There were 15 (21.13%) and 9 (18.37%) TMB-high patients in each cohort. The mutational landscapes of these cross-ancestry cohorts were disparate. The top 5 frequently mutated genes significantly differed between the Chinese (EGFR = 40.85%, MUC16 = 21.13%, MUC5B = 15.49%, MUC17 = 14.08%, CSMD3 = 12.68%) and TCGA (TP53 = 51.02%, LRP1B = 36.73%, RYR2 = 36.73%, TTN = 36.73%, MUC16 = 34.69%) cohorts. There were higher proportions of the EGFR (40.85% vs. 14.29%, *p* < 0.01) mutant type but lower proportions of TP53 (9.86% vs. 51.02%, *p* < 0.01) and KRAS (4.23% vs. 20.41%, *p* = 0.01) mutant types in the TMUCIH-LUAD cohort. However, no significant difference of TMB was found between the two cohorts before (*p* = 0.11) and after dichotomization (*p* = 0.89). Detailed diagrams of patient selection and genomic landscapes of these final cohorts are presented in Figure 1.

There was no statistical difference in baseline variables between the TMB-high and TMB-low groups in the TMUCIH-LUAD cohort, whereas T-stage indicated statistical differences in TCGA-LUAD cohort (*p* = 0.03) for a higher ratio of advanced T stages among TMB-high patients. Age and N stage (*p* < 0.01), but not other baseline variables, such as sex, side and lobe of tumor, attenuation, and the T and M stages, revealed statistical differences which suggested that it was relatively fair to compare the performance of rMB in two cohorts. The detailed comparison of the baseline variables is presented in Table 1.

**TABLE 1:** Comparison of TMB and baseline variables within and between cohorts.

	TMUCIH (n = 71)			TCGA (n = 49)			*p*-value
	TMB-high	TMB-low	*p*-value	TMB-high	TMB-low	*p*-value	
Age (mean ± SD)	60.80 ± 9.08	60.96 ± 9.27	0.95	64.67 ± 9.19	66.47 ± 11.02	0.65	<0.01**
Sex (%)			1.00			0.88	0.19
Female	7 (46.67)	28 (50.00)		5 (55.56)	26 (65.00)		
Male	8 (53.33)	28 (50.00)		4 (44.44)	14 (35.00)		
Side (%)			0.58			0.69	1.00
Left	8 (53.33)	23 (41.07)		3 (33.33)	19 (47.50)		
Right	7 (46.67)	33 (58.93)		6 (66.67)	21 (52.50)		
Lobe (%)			1.00			0.24	1.00
Basal lobes	5 (33.33)	17 (30.36)		1 (11.11)	14 (35.00)		
Upper lobe	10 (66.67)	39 (69.64)		8 (88.89)	26 (65.00)		
Distribution (%)			0.72			0.22	0.21
Central	2 (13.33)	12 (21.43)		2 (22.22)	3 (7.50)		
Peripheral	13 (86.67)	44 (78.57)		7 (77.78)	37 (92.50)		
Attenuation (%)			0.19			0.06	0.31
Solid	14 (93.33)	41 (73.21)		9 (100.00)	24 (60.00)		
Sub-solid	1 (6.67)	15 (26.79)		0 (0.00)	16 (40.00)		
T-stage (%)			0.14			0.03^a^	0.77
T1	2 (13.33)	19 (33.93)		1 (11.11)	17 (42.50)		
T2	6 (40.00)	24 (42.86)		4 (44.44)	15 (37.50)		
T3	7 (46.67)	11 (19.64)		2 (22.22)	8 (20.00)		
T4	0 (0.00)	2 (3.57)		2 (22.22)	0 (0.00)		
N-stage (%)			0.13			0.38	<0.01**
N0	14 (93.33)	45 (80.36)		5 (55.56)	26 (65.00)		
N1	0 (0.00)	10 (17.86)		3 (33.33)	5 (12.50)		
N2	1 (6.67)	1 (1.79)		1 (11.11)	9 (22.50)		
M (%)			1.00			1.00	0.57
M0	15 (100.00)	55 (98.21)		9 (100.00)	38 (95.00)		
M1	0 (0.00)	1 (1.79)		0 (0.00)	2 (5.00)		
TMB (median [IQR])	12.70 [11.28,18.75]	1.90 [1.20,2.57]		14.86 [12.60,17.46]	2.67 [1.37, 4.84]		0.11
TMB group	15	56		9	40		0.89

^a^ Significant at *p* < 0.05; **significant at *p* < 0.01.

### 3.2 Development, assessment, and interpretation of rMB

The average DICE of gross tumor volumes was 0.95 ± 0.03, suggesting a consistent definition of tumoral contours and the satisfying reproducibility of segmentation between radiologists. On this basis, the ICC filter selected 1,914 radiomic features that remained robust against variations in segmentation. Subsequently, 1,017 features with either collinearity or near-zero variance were removed from the feature vector. Eventually, a total of 31 features were associated with continuous mutational counts and TMB-high simultaneously, in which first-order statistics and the Gabor filter served as the most frequent feature type and image filter. None of the features derived from the original gray-level volumes was incorporated in the final feature vector.

**FIGURE 2:**
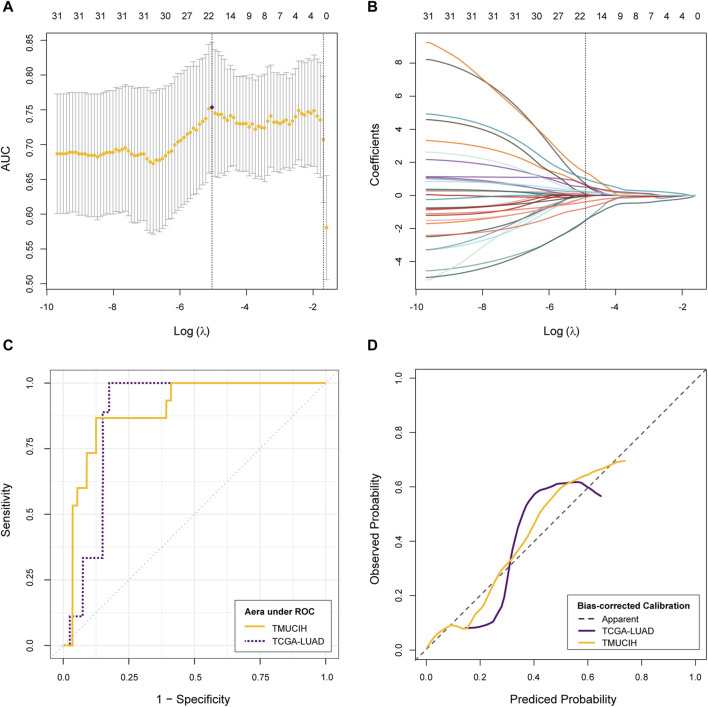
Development and validation of rMB. **(A)** Change of cross-validation metric AUCs and corresponding confidence intervals during optimizing hyper-parameter λ; **(B)** change of feature weights during LASSO–logistic classifier convergence; **(C)** evaluation of discrimination via the ROC curve; **(D)** evaluation of calibration via the calibration curve.

The LASSO–logistic classifier was parameterized with a log(λ) of −5.038 by 10-fold cross validation where a weight of 4 was attributed to TMB-high samples for the purpose of dealing with TMB imbalance. A subset of 21 features reached the highest AUC metric at 0.75 (95% CI: 0.66, 0.85) during convergence. The AUC of the purposed rMB reached 0.90 (95% CI: 0.81, 0.98, *p* < 0.01) in the discovery cohort with an accuracy of 87.32%, a sensitivity of 86.67%, and a specificity of 87.50% and 0.88 (95% CI: 0.78, 0.97, *p* < 0.01) in the validation cohort with an accuracy of 81.63%, a sensitivity of 66.67%, and a specificity of 85.00% at the same diagnostic threshold of 0.73. There is no statistical difference between the AUCs of the two cohort (D = 0.27, *p* = 0.79). The Hosmer–Lemeshow test indicated that the classifier fit well in both cohorts (*p* = 0.27 and *p* = 0.74, respectively). A summary of cross-validation, dynamic constraints of feature weights with penalty, the ROC, and calibration curves are illustrated in Figures 2A–D.

**FIGURE 3:**
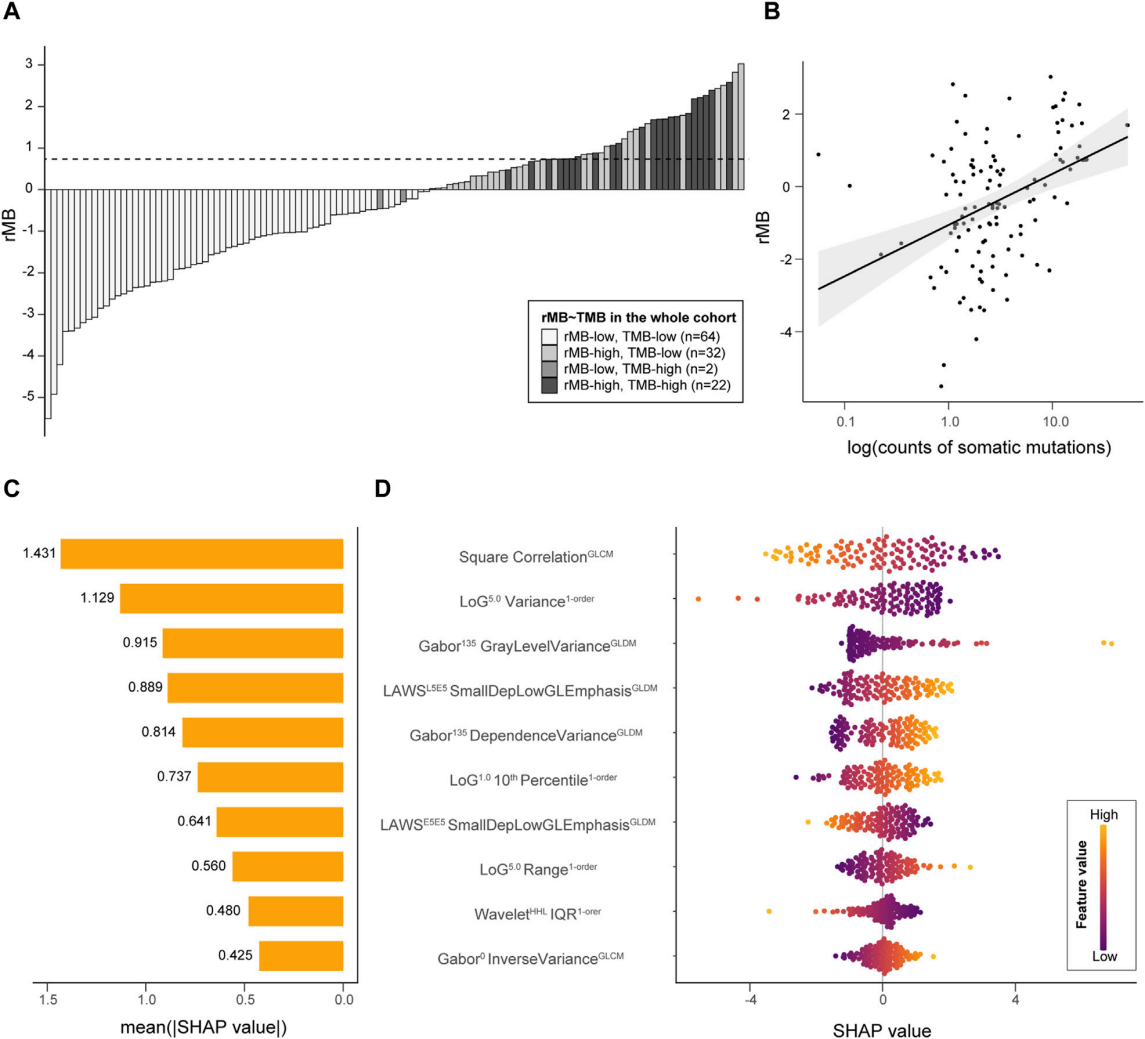
Interpretation of rMB. **(A)** A bar plot demonstrates the ordered rMB of all individuals from two cohorts; the horizontal dotted line refers to the rMB cutoff at 0.7347. **(B)** A scatter plot presents the correlation between rMB and log10 (TMB). **(C)** A bar plot reveals the importance of the top 10 radiomic features incorporated in the classifier, which are represented by the average of the Shapley value. **(D)** A bee-swarm plot shows the contribution of each sample to the predictions among the top 10 features. LoG, Laplacian of Gaussian; GLDM, gray-level dependence matrix; Dep, dependence; GL, gray level; IQR, interquartile range; E5E5, edge-like base vector of LAWS texture with a length of five elements.

TMB-high was significantly associated with increments in rMB in the discovery cohort (−0.78 ± 1.66 vs. 1.37 ± 0.88, *p* < 0.01), validation cohort (−0.87 ± 1.58 vs. 1.14 ± 0.69, *p* < 0.01), and whole cohort (−0.82 ± 1.62 vs. 1.28 ± 0.81, *p* < 0.01), as is presented in Figure 3A. In addition, a correlation between TMB and rMB was confirmed in the discovery, validation, and whole cohorts (Pearson r = 0.41, 0.41, and 0.36, respectively, all *p* < 0.01, Figure 3B). Likewise, the sum of mutational counts was also associated with rMB (negative binomial regression OR = 1.48, 1.42, and 1.43, respectively, all *p* < 0.01). The Shapley additive explanations were utilized to analyze the *post hoc* contribution of features to the rMB. The correlation of the GLCM from square filtered volume, which was negatively associated with TMB-high, served as the top feature accounting for classifier predictions (mean |SHAP| = 1.43). The top 10 contributing features implied an association that lesions with more heterogeneous radiological appearance were more likely to be TMB-high tumors. The summary plots of feature contribution are illustrated in Figures 3C,D.

### 3.3 Clinical and immune relevance of TMB and rMB

There is no difference between TMB-low and TMB-high patients in the history of malignancy and exposure to alcohol or nicotine. TMB-high was significantly associated with increased circulating monocyte percentage (5.81% ± 1.74% vs. 6.85% ± 1.54%, *p* = 0.04) and MLR (0.19 [0.14, 0.24] vs. 0.27 [0.18, 0.33], *p* = 0.01). Trends of numerical differences in counts of circulating WBCs, the lymphocyte percentage, and the SII were observed but still beyond the statistical borderline (0.05 < *p* < 0.2).

It is interesting that after regrouping patients in terms of rMB diagnostic threshold, associations between rMB-high and increments in circulating neutrophils percentage, the NLR, the dNLR, the SII, and the PLR turned up. There was also a statistical difference in circulating lymphocyte percentage between rMB levels. However, the difference in circulating monocyte percentage between rMB-low and rMB-high had narrowed such that it fell outside the significance level (5.83% ± 1.75% vs. 6.58% ± 1.63%, *p* = 0.11), albeit the significantly elevated MLR still remained in rMB-high patients. A detailed comparison of clinical variables and serum biomarkers is presented in Table 2.

**TABLE 2:** Comparison of clinical variables and serum biomarkers.

	TMB-low (n = 56)	TMB-high (n = 15)	*p*-value	rMB-low (n = 52)	rMB-high (n = 19)	*p*-value
Smoker (%)			1.00			1.00
Never	28 (50.00)	7 (46.67)		26 (50.00)	9 (47.37)	
Ever	28 (50.00)	8 (53.33)		26 (50.00)	10 (52.63)	
Package year (mean ± SD)	20.04 ± 29.35	14.97 ± 18.94	0.53	19.43 ± 29.37	17.71 ± 21.96	0.82
Alcohol (%)			0.54			1.00
Never	37 (66.07)	8 (53.33)		33 (63.46)	12 (63.16)	
Ever	19 (33.93)	7 (46.67)		19 (36.54)	7 (36.84)	
Family history of malignancy (%)			0.34			1.00
Denied	46 (82.14)	10 (66.67)		41 (78.85)	15 (78.95)	
Confirmed	10 (17.86)	5 (33.33)		11 (21.15)	4 (21.05)	
Prior/synchronous malignancy (%)			0.84			0.68
No	53 (94.64)	15 (100.0)		49 (94.23)	19 (100.0)	
Yes	3 (5.35)	0 (0.00)		3 (5.77)	0 (0.0)	
TPSA (median [IQR])	30.00 [19.24, 67.54]	32.28 [25.39, 79.66]	0.57	28.69 [19.21, 67.62]	37.31 [26.69, 73.40]	0.49
NSE (median [IQR])	11.77 [10.34, 14.16]	11.26 [10.11, 13.40]	0.81	11.17 [10.07, 13.55]	12.87 [10.98, 14.46]	0.13
CEA (median [IQR])	3.90 [2.22, 8.69]	3.87 [3.22, 12.33]	0.21	3.90 [2.29, 7.51]	3.87 [3.10, 20.51]	0.24
WBC (mean ± SD)	6.24 ± 1.32	6.99 ± 1.48	0.06	6.22 ± 1.30	6.88 ± 1.50	0.07
Neutrophils % (mean ± SD)	60.21 ± 9.17	62.43 ± 8.21	0.40	59.21 ± 8.29	64.73 ± 9.73	0.02^a^
Lymphocytes % (mean ± SD)	30.71 ± 8.27	26.98 ± 7.15	0.12	31.72 ± 7.32	25.01 ± 8.43	<0.01**
Monocytes % (mean ± SD)	5.81 ± 1.74	6.85 ± 1.54	0.04^a^	5.83 ± 1.75	6.58 ± 1.63	0.11
NLR (median [IQR])	1.86 [1.46, 2.59]	2.40 [1.99, 2.74]	0.12	1.80 [1.38, 2.44]	2.41 [2.02, 3.68]	0.01^a^
dNLR (median [IQR])	1.43 [1.14, 1.86]	1.75 [1.34, 2.04]	0.31	1.37 [1.13, 1.81]	1.75 [1.48, 2.64]	0.03^a^
PLR (median [IQR])	121.24 [95.00, 162.65]	126.92 [107.22, 178.77]	0.38	119.81 [93.13, 153.53]	143.24 [109.50, 200.44]	0.047^a^
MLR (median [IQR])	0.19 [0.14, 0.24]	0.27 [0.18, 0.33]	0.01^a^	0.18 [0.14, 0.23]	0.29 [0.18, 0.34]	<0.01**
SII (median [IQR])	436.46 [318.96, 604.53]	560.54 [431.74, 966.83]	0.16	434.89 [313.73, 557.12]	601.24 [405.55, 1,151.92]	0.01^a^
LDH (mean ± SD)	185.21 ± 32.29	176.07 ± 29.88	0.33	183.00 ± 29.41	184.05 ± 38.50	0.90

^a^ Significant at *p* < 0.05; **significant at *p* < 0.01.

Continuous variables which follow a normal distribution are presented in the format of mean ± standard deviation (SD); otherwise, they are presented as median [interquartile range (IQR)].

## 4 Discussion

In this study, we successfully developed a CT-based radiomic signature, rMB, to predict TMB-high status non-invasively for patients with lung adenocarcinoma. rMB was validated in a cross-ancestry cohort from the TCGA and presented satisfying performance of discrimination and calibration. Efforts of *post hoc* attributing variance of features to the model output were made through the SHAP approach, which implied an association between chaotic gray-level distribution and the higher possibility of TMB-high. Retrospective analysis suggested that monocytes in the peripheral blood and MTR were connected to TMB-high; however, lymphocyte-associated circulating biomarkers were more relevant to rMB-high.

The cohorts of this study were representative to some extent. The proportion of TMB-high (20% TMB ≥ 10 mut/Mb) among 120 involved LUAD patients was approximately 10%–25%, which was consistent in the results from clinical trials (McGrail et al., 2021) and cross-sectional studies (Chalmers et al., 2017). A previous study had reported disparate genomic landscape of LUAD in East Asia population with a lower median TMB of 2.04 mut/Mb (Chen et al., 2020), which suggests a more stable genome comparison with the European population. However, it is contrary that counts of mutations did not reveal any difference between the TMUCIH and TCGA-LUAD cohorts in this study, which could be ascribed to the non-random selection of participants with imaging profiles from the original TCGA-LUAD cohort, a Caucasian-predominant data set. Nevertheless, a significant difference in driver mutation was also confirmed (EGFR vs. TP53) in this study as expected. On the other hand, there was no clinical variable associated with TMB-high from our analysis. However, a history of tobacco exposure was a confirmed dose–response risk factor of higher genetic alterations in advanced-stage NSCLC (Wang et al., 2021). We blamed this inconsistency to the fact that there is a higher number of LUAD patients who were never smokers in the Asia population (Leiter et al., 2023), and distinct genomic and evolutionary characteristics of lung cancer in never-smokers were reported previously (Zhang et al., 2021). In addition, the effect of tobacco exposure on cancer genomic and derived TMB of resectable early-stage LUAD, which took up most patients (98.59%) in the TMUCIH cohort, may be weaker than it is on advanced-stage patients.

The satisfying result of this study in discriminating TMB-high LUAD patients using a machine learning-enabled radiomic signature tied well with previous studies wherein mutational load of cancer genome shapes radiological phenotypes in NSCLC. Zhang et al. reported associations between the absence of concavity, ill-defined border, less spiculation, normal adjacent bronchovascular bundle, and larger size of tumor that predict TMB-high NSCLC (Zhang et al., 2020). A recent study divided these associations into radiomic signatures of intra-tumoral and peritumoral regions, in which the former performed better in distinguishing the TMB-high group (Yang et al., 2023). Overall, these findings were in accordance with our findings with similar AUCs. Comparing our results with these studies, it must be pointed out that histological type should be considered because squamous cell carcinoma does have a higher TMB than LUAD (Chae et al., 2019). To the best of our knowledge, this study is the first investigation that reports a LUAD-dedicated imaging biomarker for preoperative TMB stratification. A further attempt that used convolutional neural network, a representative algorithm of deep learning, to predict TMB status provided a comparable performance [AUC of test set: 0.81 (0.77,0.85)] in a larger Chinese NSCLC cohort (He et al., 2020). However, the class activation map shifted out of the contour of the tumor, which may indicate the contribution of the peritumoral region or a somewhat overfitting of the model. Leveraging the classic intra-tumoral radiomic approach, the precise correlation between TMB and radiological phenotypes could be established without the concern of spatial factors.

The *post hoc* analysis of immune biomarkers revealed that a proportion of monocytes in the peripheral blood and derived MLR were associated with TMB-high. This could imply that immunogenicity of the tumor is driven by neoantigen, a downstream effect of increased genomic alterations (Haddad et al., 2022), which mobilizes circulating monocytes infiltrating the tumor to play the role of regulators in tumor microenvironments. A previous finding has suggested that circulating CD14 (+)CD16(−)HLA-DR(hi) monocytes could predict benefits of immunotherapy in melanoma (Krieg et al., 2018). There has also been encouraging evidence that emphasizes the link between enriched tumor monocytes and immunochemotherapy outcomes in esophageal adenocarcinoma (Carroll et al., 2023). On the flip side, when regrouping patients in terms of the rMB levels, biomarkers relevant to lymphocytes and the SII accounted for the variance in radiological signals instead of those relevant to monocytes. We believe that such a conversion may be associated with the restriction of spatial attention on the primary tumor site because tumor-infiltrating lymphocytes and cytotoxic killing induced by CD8(+) T cells serve as the last effective factor in neoantigen-induced antitumor immunity (Gueguen et al., 2021). Moreover, these results highlight that little is known about the relationship between radiological phenotypes and the mononuclear phagocytic system, as well as their interaction with adaptive immune resistance at the tumor site and through circulation.

Our study does have some limitations. First, the small sample size with a lack of clinical and biomarker information in the TCGA cohort weakens the power of predictive model and rMB performance, and the candidate set of discriminative features may differ from our study where local optima may conceal the real patterns in these cross-scale data. A multicenter cooperation is expected to validate our insights in a larger cohort. Second, the mixture of contrast-enhanced studies may lead to potential bias even if standardization and rescaling of original image, feature vectors, and ComBat harmonization were taken to compensate for such a confounding effect. The use of contrast-enhanced images may guide the model to magnify a specific histological feature of a tumor such as angiogenesis. A further comparison or pathological contrast would help in isolating the impact of such factors. Moreover, the correlation among TMB, rMB, predicted neoantigens, and tumor-infiltrating immune cells ought to be further assessed. Finally, the performance of rMB in guiding the application of immune checkpoint inhibitors should be tested in a real-world data set with survival outcomes.

In conclusion, the intra-tumor radiomic signature could predict lung adenocarcinoma patients with higher TMB. Insights from SHAP interpretation may enhance the persuasiveness of the purposed signature for further clinical application. rMB would be a promising tool to triage patients who might benefit from an NGS test.

## Data Availability

The raw data supporting the conclusions of this article will be made available by the authors, without undue reservation.
