# Supplementary material for: Development and validation of an interpretable radiomic signature for preoperative estimation of tumor mutational burden in lung adenocarcinoma
Source: Front Genet. 2024 Apr 10;15:1367434. doi: 10.3389/fgene.2024.1367434 (PMC11039798; doi:10.3389/fgene.2024.1367434)
Supplement: Supplementary file 1 [file DataSheet2.PDF]

```
# Figure1
# --Fig1.B and Fig1.D
library(maftools)
library(ggsci)
maf <- read.maf("path of a integrated WES maf") # real path is anonymized
col_pal <- ggsci::pal_lancet()(8)
oncoplot(maf, top=20, colors=col_pal)

# Figure 2
library(glmnet)
library(ggsci)
library(precrec)
require(ggplot2)

cvmodel <-"an fitted object from function 'cv.glmnet'"
radscore <-"a linear combination of variables after selection and their
weights"
label <-"a factor of ground truth with two levels 0/1 for binary
classification"
# --Fig2.A
plot(cvmodel)
# --Fig2.B
plot(cvmodel$glmnet.fit, xvar="lambda", col=pal_futurama(alpha=0.75)(12),
lw=2)
# --Fig2.C
roccurve <- evalmod(scores = as.numeric(radscore),
                    labels = as.numeric(label))
rocplot <- autoplot(roccurve,"roc")
# --Fig2.D
plot.CalibrationCurveLogit <- function(radscore,
                                        true_label,
                                        nbins = 10){

require(predtools);require(ggplot2);require(ResourceSelection);require(rms)

    rs <- radscore
    label <-  true_label #only accept a factor with level 0 and 1
    df <-  data.frame(label = label, radscore = rs)
    fit <- glm(label~radscore, data = df, family = binomial(link="logit"))

    # H-L test
    hl.test <- hoslem.test(fit$y, fitted(fit), g = nbins)
    print(hl.test)

    # Calibration Curve
    fit2 <- lrm(label~radscore, data = df, x=TRUE, y=TRUE)
    cal <- calibrate(fit2, method='boot', B=500) #-> a named matrix

    #Plot
    plot(cal, xlim=c(0,1), ylim=c(0,1),
         xlab = "Prediced Probability", ylab = "Observed Probability",
         cex.lab=1, cex.axis=1, cex.main=1, cex.sub=1, legend=FALSE) +
        abline(0,1, lty = 3,lwd = 2, col = "#000000") +
        lines(cal[,c(1,3)], type = 'l', lwd = 3,pch = 16, col = "#2166AC")
+
        legend(0.7, 0.2, c("Apparent","Bias-corrected","Ideal"),
               lty = c(2,1),
               lwd = c(2,3),
```

```
                col = c("#000000","#2166AC"),
                bty = "n"
         )
    plt <- recordPlot()

    return(list(calibration = fit2, plot.cali = plt, test=hl.test))
}

# Figure 3
library(ggplot2)
# --Fig3.A
df <- rbind("prediction.train", "prediction.test") # original data is
anonymized
df <- df[order(df$radscore),]
df$idx <- seq(1,nrow(df))
df$color <- (df$true_label-1)*2 + df$predicted_label -1 # Encode gray
levels
plt <- ggplot(data=df, aes(x=idx, y=radscore, fill=color)) +
    geom_bar(stat = "identity", color="black", width=1, size=0.3) +
    theme_classic()  +
    geom_hline(yintercept = 0.734670158955174, linetype="dashed") +
#cutoff=0.73
    scale_fill_brewer(palette = "Greys") +
    scale_y_continuous(breaks = seq(-6,4,1))

# --Fig3.B
df <- data.frame(TMB = "all TMB", rMB = "all rMB") # original data is
anonymized
plt <- ggplot(df, aes(x=TMB , y=rMB))
plt + scale_x_log10() +
    geom_point() +
    geom_smooth(method = "lm", color="black", fill="lightgray") +
    labs(x="log(counts of somatic mutations)", y="rMB")
    theme_classic()
# --Fig3.C
library(shapviz)
library(kernelshap)
sp <- shapviz(kernelshap(cvmodel$glmnet.fit,
                         pred_fun = glmnet::predict.glmnet,
                         s = cvmodel$lambda.min,
                         X = as.matrix(data),
                         bg_X = as.matrix(data)))
sv_importance(sp, kind="bar", max_display = 10, show_numbers = TRUE) +
    theme_classic() + scale_y_discrete(position = "right") +
scale_x_reverse()
# --Fig3.D
sv_importance(sp, kind="beeswarm", max_display = 10) + theme_classic()
```
